# Supplementary material for: Parental Feeding Styles and Risk of a New Carious Lesion in Preschool Children: A Longitudinal Study
Source: Nutrients. 2023 Oct 16;15(20):4387. doi: 10.3390/nu15204387 (PMC10609665; doi:10.3390/nu15204387)
Supplement: Supplementary file 1 [file nutrients-15-04387-s001.zip › nutrients-2649207-supplementary.pdf]

Supplementary Table S1. Parental Feeding Style Questionnaire.

Tip: This questionnaire is to investigate the style you feed your child, please tick (√) the corresponding box.

(a) Instrumental feeding

Item	Never	Rarely	Sometimes	Often	Always
In order to get my child to behave him/herself I promise him/her something to eat.					
If my child misbehaves I withhold his/her favourite food.					
I use puddings as a bribe to get my child to eat his/her main course.					
I reward my child with something to eat when s/he is well behaved.					

(b) Emotional feeding

Item	Never	Rarely	Sometimes	Often	Always
I give my child something to eat to make him/her feel better when s/he is feeling upset.					
I give my child something to eat to make him/her feel better when s/he has been hurt.					
I give my child something to eat if s/he is feeling bored.					
I give my child something to eat to make him/her feel better when s/he is worried.					
I give my child something to eat to make him/her feel better when s/he is feeling angry.					

(c) Promoting and encouragement to eat

Item	Never	Rarely	Sometimes	Often	Always
I encourage my child to look forward to the meal.					
I praise my child if s/he eats what I give him/her.					
I encourage my child to eat a wide variety of foods.					
I present food in an attractive way to my child.					
I encourage my child to taste each of the foods I serve at mealtimes.					
I encourage my child to try foods that s/he hasn't tasted before.					
I encourage my child to enjoy his/her food.					
I praise my child if s/he eats a new food.					

(d) Control over eating

Item	Never	Rarely	Sometimes	Often	Always
I decide when it is time for my child to have a snack.					
I decide how many snacks my child should have.					
I decide what my child eats between meals.					
I decide the times when my child eats his/her meals.					
I insist my child eats meals at the table.					
I allow my child to choose which foods to have for meals*.					
I allow my child to wander around during a meal*.					
I allow my child to decide when s/he has had enough snacks to eat*.					
I let my child eat between meals whenever s/he wants*.					
I let my child decide when s/he would like to have her meal*.					

*Reversed questions.

Supplementary Table S2. Parental feeding styles and oral health-related behaviors of the participants according to baseline caries status (n=1181).

	**Caries-free (n=539)**	**Caries (n=642)**	***p***
	**Mean (SD)**	**Mean (SD)**	
**Parental feeding style**			
Instrumental feeding	2.54 (0.54)	2.62 (0.63)	**0.022**[a]
Emotional feeding	2.23 (0.66)	2.31 (0.68)	**0.042**[a]
Prompting or encouragement to eat	4.00 (0.64)	3.81 (0.71)	**< 0.001**[a]
Control over eating	3.76 (0.45)	3.59 (0.48)	**< 0.001**[a]
**Visible plaque index**[d]	0.34 (0.19)	0.43 (0.18)	**< 0.001**[a]
	**n (%)**	**n (%)**	
**Frequency of eating deserts**			**0.001**[c]
≥2 time per week	306 (42.3%)	418 (57.7%)	
1 time per week	103 (47.7%)	113 (52.3%)	
<1 time per week	130 (53.9%)	111 (46.1%)	
**Frequency of drinking sugar-sweetened beverages**			**< 0.001**[c]
≥2 time per week	25 (26.3%)	70 (73.7%)	
1 time per week	50 (35.5%)	91 (64.5%)	
<1 time per week	464 (49.1%)	481 (50.9%)	
**Consumption of sweets before sleep**			**< 0.001**[c]
Frequently	80 (40.4%)	118 (59.6%)	
Occasionally	198 (39.8%)	299 (60.2%)	
Never	261 (53.7%)	225 (46.3%)	
**Frequency of toothbrushing**			**0.009**[b]
≥2 times per day	266 (49.8%)	268 (50.2%)	
<2 times per day	273 (42.2%)	374 (57.8%)	
**Supervised toothbrushing**			**0.005**[b]
Frequently	296 (49.7%)	300 (50.3%)	
Occasionally or never	243 (41.5%)	342 (58.5%)	
**Using fluoride toothpaste**			0.371[b]
Yes	275 (47.1%)	309 (52.9%)	
No	152 (46.1%)	178 (53.9%)	
Unknown	112 (41.9%)	155 (58.1%)	
**Professional fluoride application within 6 months**			0.063[b]
Yes	138 (43.1%)	182 (56.9%)	
No	360 (47.9%)	391 (52.1%)	
Unknown	41 (37.3%)	69 (62.7%)	

SD: standard deviation. [a] Student's t test. [b] Chi-square test. [c] Linear-by-linear association tests. [d] Visible plaque index was calculated as a percentage of the number of surfaces with plaque to the total number of surfaces examined. *p*-value in bold indicated statistical significance.

Supplementary Table S3. Demographic characteristics of the participants according to baseline caries status (n=1181).

	**Caries-free (n=539)**	**Caries (n=642)**	***p***
	**Mean (SD)**	**Mean (SD)**	
**Age (months)**	43.81 (3.65)	44.88 (3.44)	**< 0.001**[a]
**BMI**	15.00 (1.22)	14.95 (1.20)	0.483[a]
	**n (%)**	**n (%)**	
**Gender**			0.970[b]
Male	281 (45.7%)	334 (54.3%)	
Female	258 (45.6%)	308 (54.4%)	
**Residence**			**< 0.001**[b]
Urban	355 (55.6%)	283 (44.4%)	
Suburban	184 (33.9%)	359 (66.1%)	
**Paternal education level**[d]			**< 0.001**[b]
College or above	459 (50.3%)	453 (49.7%)	
High school or below	79 (29.5%)	189 (70.5%)	
**Maternal education level**[e]			**< 0.001**[b]
College or above	470 (50.7%)	457 (49.3%)	
High school or below	67 (26.9%)	182 (73.1%)	
**Household monthly income**[f]			**< 0.001**[c]
High-income	211 (51.7%)	197 (48.3%)	
Moderate-income	149 (47.0%)	168 (53.0%)	
Low-income	169 (38.7%)	268 (61.3%)	

SD: standard deviation. [a] Student's t test. [b] Chi-square test. [c] Linear-by-linear association tests. [d] 1 missing data, [e] 5 missing data, [f] 19 missing data. *p*-value in bold indicated statistical significance.

Supplementary Table S4. Parental feeding styles and oral health-related behaviors of the participants according to the change in dmft after 15 months (n=1090).

	No increase in dmft, Δdmft=0 (n=449)	Increase in dmft, Δdmft>0 (n=641)	*p*
	**Mean (SD)**	**Mean (SD)**	
**Parental feeding style**			
Instrumental feeding	2.57 (0.55)	2.61 (0.61)	0.223[a]
Emotional feeding	2.24 (0.66)	2.30 (0.69)	0.181[a]
Prompting or encouragement to eat	3.99 (0.63)	3.84 (0.72)	**0.001[a]**
Control over eating	3.75 (0.45)	3.61 (0.48)	**< 0.001[a]**
**Visible plaque index[d]**	0.34 (0.19)	0.42 (0.18)	**< 0.001[a]**
	**n (%)**	**n (%)**	
**Past caries experience**			**< 0.001[b]**
Caries-free	319 (64.4%)	176 (35.6%)	
Caries	130 (21.8%)	465 (78.2%)	
**Frequency of eating deserts**			**0.007[c]**
⩾2 time per week	252 (37.3%)	424 (62.7%)	
1 time per week	99 (50.3%)	98 (49.7%)	
<1 time per week	98 (45.2%)	119 (54.8%)	
**Frequency of drinking sugar-sweetened beverages**			**0.005[c]**
⩾2 time per week	27 (30.3%)	62 (69.7%)	
1 time per week	47 (35.1%)	87 (64.9%)	
<1 time per week	375 (43.3%)	492 (56.7%)	
**Consumption of sweets before sleep**			**0.021[c]**
Frequently	73 (39.9%)	110 (60.1%)	
Occasionally	166 (36.3%)	291 (63.7%)	
Never	210 (46.7%)	240 (53.3%)	
**Frequency of toothbrushing**			**< 0.001[b]**
≥2 times per day	232 (47.3%)	259 (52.7%)	
<2 times per day	217 (36.2%)	382 (63.8%)	
**Supervised toothbrushing**			**< 0.001[b]**
Frequently	259 (47.4%)	287 (52.6%)	
Occasionally or never	190 (34.9%)	354 (65.1%)	
**Using fluoride toothpaste**			0.817[b]
Yes	229 (42.1%)	315 (57.9%)	
No	122 (40.7%)	178 (59.3%)	
Unknown	98 (39.8%)	148 (60.2%)	
**Professional fluoride application within 6 months**			**0.039[b]**
Yes	120 (40.8%)	174 (59.2%)	
No	299 (43.0%)	396 (57.0%)	
Unknown	30 (29.7%)	71 (70.3%)	

SD: standard deviation. [a] Student's t test. [b] Chi-square test. [c] Linear-by-linear association tests. [d] Visible plaque index was calculated as a percentage of the number of surfaces with plaque to the total number of surfaces examined. *p*-value in bold indicated statistical significance.

Supplementary Table S5. Demographic characteristics of the participants according to the change in dmft after 15 months (n=1090).

	**No increase in dmft, Δdmft=0 (n=449)**	**Increase in dmft, Δdmft>0 (n=641)**	***p***
	**Mean (SD)**	**Mean (SD)**	
**Age (months)**	43.89 (3.61)	44.83 (3.44)	**< 0.001**[a]
**BMI**	14.98 (1.25)	14.96 (1.18)	0.774[a]
	**n (%)**	**n (%)**	
**Gender**			0.769[b]
Male	232 (40.8%)	337 (59.2%)	
Female	217 (41.7%)	304 (58.3%)	
**Residence**			**< 0.001**[b]
Urban	285 (48.0%)	309 (52.0%)	
Suburban	164 (33.1%)	332 (66.9%)	
**Paternal education level**[d]			**< 0.001**[b]
College or above	379 (44.6%)	471 (55.4%)	
High school or below	70 (29.3%)	169 (70.7%)	
**Maternal education level**[e]			**0.001**[b]
College or above	377 (43.9%)	481 (56.1%)	
High school or below	71 (31.3%)	156 (68.7%)	
**Household monthly income**[f]			**0.008**[c]
High-income	176 (46.6%)	202 (53.4%)	
Moderate-income	119 (40.5%)	175 (59.5%)	
Low-income	151 (37.2%)	255 (62.8%)	

SD: standard deviation. [a] Student's t test. [b] Chi-square test. [c] Linear-by-linear association tests. [d] 1 missing data, [e] 5 missing data, [f] 12 missing data. *p*-value in bold indicated statistical significance.
